# Invasive Candidiasis in Adult Patients with COVID-19: Results of a Multicenter Study in St. Petersburg, Russia

**DOI:** 10.3390/jof9090927

**Published:** 2023-09-14

**Authors:** Olga Kozlova, Ekaterina Burygina, Sofya Khostelidi, Olga Shadrivova, Andrey Saturnov, Denis Gusev, Aleksandr Rysev, Anatoliy Zavrazhnov, Maria Vashukova, Galina Pichugina, Mikhail Mitichkin, Sergey Kovyrshin, Tatiana Bogomolova, Yulia Borzova, Ellina Oganesyan, Natalya Vasilyeva, Nikolay Klimko, Working Group

**Affiliations:** 1 Kashkin Research Institute of Medical Mycology; North-Western State Medical University Named after I.I. Mechnikov, 191015 Saint-Petersburg, Russia; ekarina.burygina@szgmu.ru (E.B.); sofianic@mail.ru (S.K.); olshadr@mail.ru (O.S.); sergei.kovyrshin@szgmu.ru (S.K.); bogomol52@list.ru (T.B.); borzova-y@mail.ru (Y.B.); ellina.oganesyan@gmail.com (E.O.); natalya.vasileva@szgmu.ru (N.V.); n_klimko@mail.ru (N.K.); 2 Leningrad Regional Hospital, 194291 Saint-Petersburg, Russia; saturn-07@yandex.ru; 3 Botkin’s Hospital, 195067 Saint-Petersburg, Russia; gusevden-70@mail.ru (D.G.); mavashukova@yahoo.com (M.V.); 4 Saint Petersburg Research Institute of Emergency Medicine n.a. I.I. Dzhanelidze, 192242 Saint-Petersburg, Russia; rysev.aleksander2015@yandex.ru (A.R.); gal-gal2000@mail.ru (G.P.); 5 City Mariinskaya Hospital, 191014 Saint-Petersburg, Russia; zaa.70@mail.ru (A.Z.); meg657030@mail.ru (M.M.)

**Keywords:** yeast, fungal, mycoses, SARS-CoV-2, acute respiratory distress syndrome, *Candida*, candidiasis, COVID-19

## Abstract

We studied the risk factors, etiology, clinical manifestations, and treatment outcomes of COVID-19-associated invasive candidiasis (COVID-IC) in adult patients admitted to six medical facilities in St. Petersburg. (November 2020–December 2022). In this retrospective study, we included 72 patients with COVID-IC with a median age of 61 years (range 29–96), 51% of whom were women. The predisposing factors for COVID-IC were a central venous catheter (CVC) for more than 10 days (the odds ratio (OR) = 70 [15–309]), abdominal surgical treatment performed in the previous 2 weeks (OR = 8.8 [1.9–40.3]), bacteremia (OR = 10.6 [4.8–23.3]), pulmonary ventilation (OR = 12.9 [5.9–28.4]), and hemodialysis (OR = 11.5 [2.5–50.8]). The signs and symptoms of COVID-IC were non-specific: fever (59%), renal failure (33%), liver failure (23%), and cardiovascular failure (10%). *Candida albicans* (41%) predominated among the pathogens of the candidemia. The multidrug-resistant *Candida* species *C. auris* (23%) and *C. glabrata* (5%) were also identified. Empirical therapy was used in 21% of COVID-IC patients: azole-93%, echinocandin–7%. The majority of COVID-IC patients (79%) received, after laboratory confirmation of the diagnosis of IC, fluconazole (47%), voriconazole (25%), echinocandin (26%), and amphotericin B (2)%. The 30 days overall survival rate was 45%. The prognosis worsened concomitant bacteremia, hemodialysis, and long-term therapy by systemic glucocorticosteroids (SGCs), bronchial colonization with *Candida* spp. The survival prognosis was improved by the early change/replacement of CVC (within 24 h), the initiation of empirical therapy, and the use of echinocandin. Conclusions: We highlighted the risk factors that predispose COVID-19 patients to candidiasis and worsen the survival prognosis. Their individual effects in patients with COVID-19 must be well understood to prevent the development of opportunistic co-infections that drastically lower chances of survival.

## 1. Introduction

The pandemic of COVID-19 significantly increased the number of patients with a prolonged length of stay in the intensive care unit (ICU) [1,2]. Before the pandemic of COVID-19, *Candida* spp. accounted for 8.4% of the pathogens of nosocomial infections in the large hospitals of the Russian Federation. [3]. The frequency of invasive mycoses, including invasive candidiasis (IC), in the patients with COVID-19 varied depending on the country and region. According to international publications, the frequency of IC in patients with COVID-19 in Spain was 0.7–23.5% [4], in India—2.5% [5], in Italy—8% [6], in China—23.5% [7], and in the USA—25.5% [8]. Patients with severe COVID-19 often had multiple risk factors for the development of invasive candidiasis (IC): the long-term use of broad-spectrum antibacterial drugs and systemic glucocorticosteroids (GCS), the use of central venous catheters (CVC), and parenteral nutrition, prolonged neutropenia, and lymphocytopenia [8]. It was shown that IC significantly increased mortality in critically ill patients with COVID-19 (46%) which is presumably higher than in severely ill patients with COVID-19 without IC (25.8%) [9]. When COVID-19-associated invasive candidiasis (COVID-IC) occurred, the probability of death during hospitalization was doubled, the duration of treatment was prolonged for 3–30 days, and the cost of treatment was increased by 1.5–5 times [10]. The data regarding the frequency of IC among the patients with COVID-19, risk factors, etiological agents, and the effectiveness of therapy in the Russian Federation were lacking. 

The aim of our study was to assess the risk factors, etiology, clinical manifestations, and treatment outcomes of COVID-IC in adult patients in the hospitals in St. Petersburg.

## 2. Materials and Methods

This retrospective multicenter study was conducted in the six hospitals in St. Petersburg, Russia (November 2020–December 2022). The study protocol was approved by the Local Ethic Committee of the North-Western State Medical University, named after I.I. Mechnikov (protocol code 4, 12 April 2023). The study group included 72 patients with COVID-IC, median age—61 years (29–96), males—49%. Control group I—75 patients with COVID-19 without IC, median age—63 years (29–89), males—49%. Control group II—78 patients with IC without COVID-19, median age—60.5 years (37–91), males—51%. Risk factors, comorbidities, clinical features of the disease course, treatment outcomes, and survival rate were analyzed.

The Sequential Organ Failure Assessment (SOFA) scores were calculated to assess the severity of disease and to predict the clinical outcomes. This tool was based on six criteria (score ranges from 0 to 4 for each) reflecting the function of the organ systems (respiratory, cardiovascular, renal, neurological, hepatic, and coagulation). The total score ranged 0–24. The higher the score, the greater the insufficiency of the assessed systems and the degree of multiorgan dysfunction.

The diagnosis of COVID-19 was carried out according to the criteria presented in the “Prevention, diagnosis and treatment of new coronavirus infection (COVID-19)” temporary guidelines of the Ministry of Health of the Russian Federation [2]. COVID-19 was verified by a positive SARS-CoV-2 polymerase chain reaction (PCR) from nasal and/or pharyngeal swabs or the presence of CT scan features typical of COVID-19 with a positive test for antibodies (Ig M) to SARS-CoV-2 [2].

Invasive candidiasis was diagnosed according to the criteria of the European Organization for Research and Treatment of Cancer/Mycoses Study Group Education and Research Consortium (EORTC/MSGERG) 2020 [11]. The diagnosis of IC was proven by the detection of one or more *Candida* species in normally sterile biological specimens: blood, peritoneal, and pericardial fluid (via direct puncture).

Microscopic examinations and the identification of the *Candida* species culture were carried out in the affiliated microbiological laboratories. The blood samples (20–30 mL) were obtained twice a day for three days via direct venipuncture from different points and then incubated in an automatic analyzer for hemocultures (BACTEC, “Becton Dickinson”, Franklin Lakes, NJ, USA). When signs of fungal growth were detected, the isolated *Candida* spp. colonies were transferred on Sabouraud agar with chloramphenicol (BioMerieux, Marcy l’Etoile, France). The duration of incubation was at least 5 days at temperature +37 °C to obtain growth of fungi. The species identification was confirmed via MALDI-TOF (matrix-assisted laser desorbtion ionization time-of-flight mass spectrometry) mass spectrometry. All isolates of *Candida* spp. were tested for sensitivity to antifungal drugs (amphotericin B, voriconazole, fluconazole, caspofungin, and micafungin). The indicative criteria recommended by the CDC (Center for Control and Prevention of Infectious Diseases, USA, URL: https://www.cdc.gov/fungal/candida-auris/c-auris-antifungal.html, accessed on 22 December 2022) were used to interpret the obtained values of minimum inhibitory concentrations (MIC). The sensitivity of *Candida auris* was determined using Sensititre YeastOneYO10 colorimetric panels (ThermoFisher Scientific, Renfrew, UK) in accordance with the manufacturer’s instructions. The sensitivity of Candida non-auris was tested with serial dilutions relative to MIC values determined according to the “The European Committee on Antimicrobial Susceptibility Testing. An overview of antifungal ECOFFs and clinical breakpoints for yeasts, molds, and dermatophytes was obtained using the EUCAST E.Def 7.3, E.Def 9.4 and E.Def 11.0 procedures (version 3, 2022)” [12].

The obtained biomedical data were processed using the STATISTICA for Windows software system (version 13.0). Demographic and clinical characteristics of patients were presented with number and percentage for categorical variables and median (Me) and interquartile range (Q1–Q3) for continuous variables. The groups were compared using the Mann–Whitney U test for continuous variables and using Pearson’s chi-squared test for categorical variables. The odds ratio (OR) with a 95% confidence interval (CI) was used to assess risk factors. The factor was considered significant if OR > 1. The 30-day survival of patients in the main group and the effects of various risk factors on survival were evaluated via the Kaplan–Meier curve. The differences were considered significant if *p* < 0.05.

## 3. Results

### 3.1. Demographic Data

The control and study groups were ascertained and comparable by number of patients, age, and gender (Table 1). Based on the analysis of the database, concomitant diseases such as coronary heart disease (CHD), peptic ulcer, oncological diseases, hepatitis, tuberculosis, autoimmune diseases, decompensation of diabetes mellitus (based on the conclusions of specialists), severe viral infections, injuries, renal, hepatic, and heart failure were taken into account.

The median period from the detection of SARS-CoV-2 to the diagnosis of COVID-IC was 16 (1–31) days (range 0–52 days); from the admission to the hospital to the diagnosis of COVID-IC, it was 20 (1–39.2) days (range 10–83 days); from the admission to the ICU to the diagnosis of COVID-IC, it was 15 (2–28) days (range 5–63 days)**.** The development of COVID-IC was accompanied by a significant increase in the length of stay in the hospital (median–44 vs. 22 days) and ICU (18 vs. 4 days) compared to the group of patients with COVID-19 without IC.

### 3.2. Factors That Predispose Candidiasis in COVID-19 Patients

**Figure 1:**
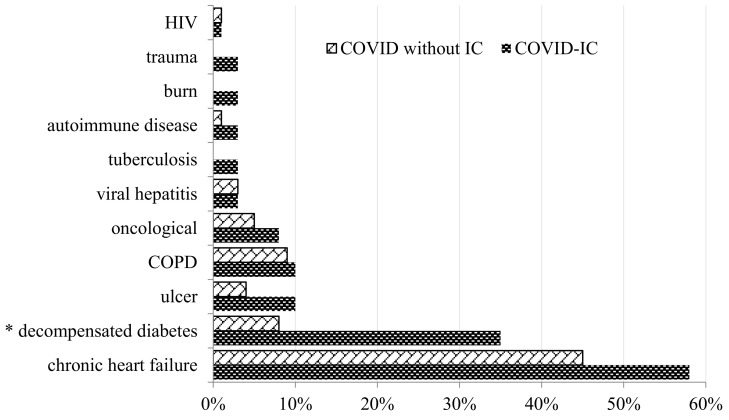
Frequency of comorbidities in the COVID-IC and COVID without IC groups (* *p* < 0.0001). HIV—human immunodeficiency virus; COPD—chronic obstructive pulmonary disease.

The patients with COVID-IC compared to the patients with COVID without IC had a higher prevalence of decompensated diabetes mellitus (35% vs. 8%, *p* < 0.0001), and there was no significant difference between the two groups for the other comorbidities (Figure 1).

**Figure 2:**
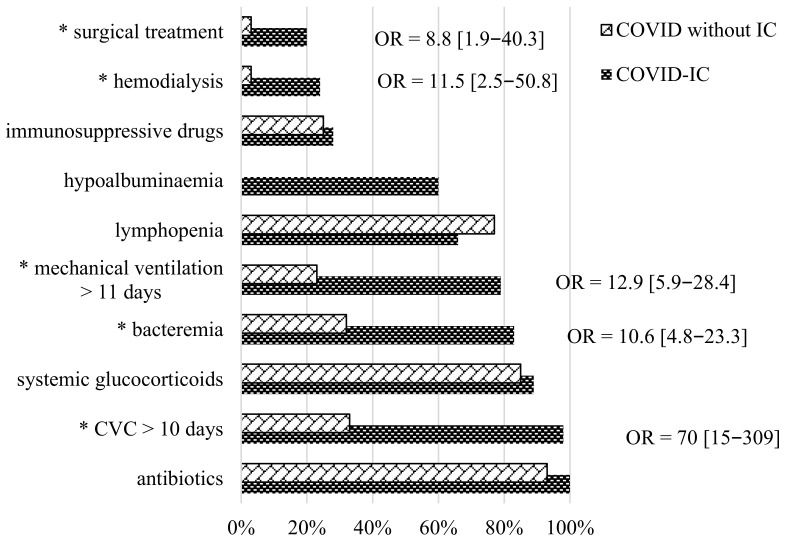
Risk factors for COVID-IC (* OR > 1).

The assessment of the risk factors of the disease was one of the important aspects of the diagnostic search. Based on the analyzed data, the frequency of various COVID-IC risk factors was studied between the COVID-IC and COVID-19 without IC groups (Figure 2).

A comparative analysis of the results showed that the probability of COVID-IC development significantly increased in patients with prolonged (median 10 days) use of a central venous catheter (CVC), abdominal surgical treatment performed in the previous 2 weeks, bacteremia, prolonged (median 11 (1–21) days) artificial lung ventilation, and hemodialysis. The CVC was widely used in the patients with COVID-19 in the ICU. The duration of CVC placement in our patients ranged from 0 to 39 days (median 10 days) before the development of IC. After the IC was diagnosed, the CVC was removed or replaced in the period from 0 to 6 days (median 24 h). Previous or concomitant bacteremia was detected in patients with COVID-IC in 83% vs. 32% compared to the patients with COVID without IC.

In 45% of cases, a polymicrobial infection was identified. The most common pathogens were *Staphylococcus* spp. (45%), *Klebsiella pneumoniae* (33%), *Acinetobacter* spp. (25%), *Proteus mirabilis* (12%), *Pseudomonas aeruginosa* (12%), *Corynebacterium* spp. (8%), *Enterococcus* spp. (6%), and in the single cases: *Trichosporon asahii, Providesia smartii,* and *Micrococcus luteus.* Comparing the COVID-IC and COVID without IC groups, artificial lung ventilation was used in 79% vs. 23% of cases. The duration of the ventilation was 0–39 days (median 11 (1–21) days) before the IC occurred. Colonization of the bronchial tree mucosa by *Candida* spp. was observed in 33% of patients from the study group. All patients were administered antibacterial therapy, and most of them received two or more antibacterial drugs at the time of the development of IC. In most cases, it was vancomycin or meropenem. Systemic glucocorticosteroids (SGCs) were frequent risk factors in both the study and COVID without IC control groups—89% vs. 85%. The doses of SGCs in both groups were comparable and high: in prednisone-equivalent doses, 0.5–4 mg/kg/day (median 1.65 (0.5–2.65 mg/kg/day)). The average number of days of SGCs treatment in the study group was 4–45 (median 10) days. The frequency of lymphocytopenia in the patients with COVID-IC and COVID without IC was 66% vs. 77% (*p* = 0.041), respectively. The level of lymphocytes in patients with COVID-IC was 0.25–3.00 × 10^9^/L (median 0.70 × 10^9^/L).

### 3.3. Clinical Manifestations of COVID-IC

**Figure 3:**
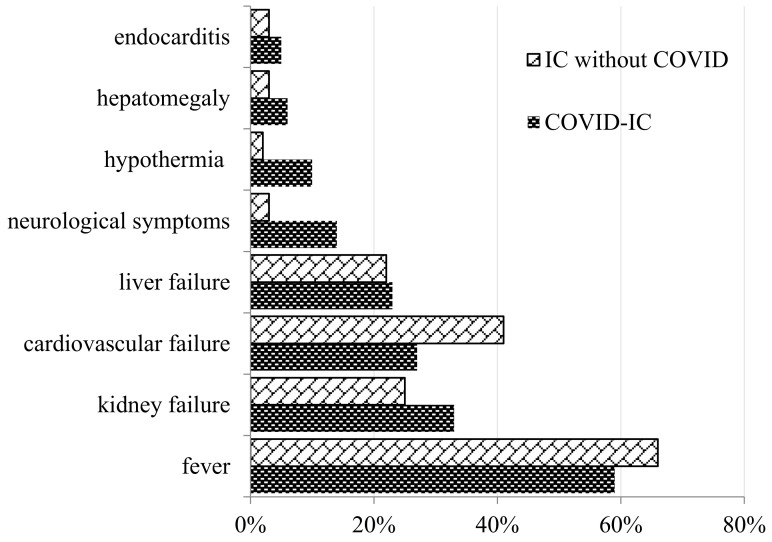
Clinical manifestations of COVID-IC.

We compared the clinical manifestations in COVID-IC and IC without COVID groups of patients. It should be noted that patients with COVID-19 at the time of the development of IC had a higher SOFA score. The SOFA index was 8 vs. 6.5 points in comparison with the patients from the control group. The clinical manifestations of COVID-IC were nonspecific. An increased body temperature despite antibacterial treatment was noted in 59% of patients. In the study and control groups, the development of renal (33% vs. 25%) and hepatic (23% vs. 22%) failure was frequently noted. Cardiovascular failure developed more often (10% vs. 2%) in patients with COVID-IC (Figure 3).

### 3.4. Etiology of COVID-IC

The diagnosis of invasive candidiasis (IC) was based on the isolation of *Candida* spp. from normally sterile biological specimens: blood, peritoneal, and pericardial fluid (via direct puncture). The pathogens of COVID-IC (Table 2) were *C. albicans* (41%), *C. auris* (23%), *C. parapsilosis* (8%), *C. guilliermondi* (7%), *C. glabrata* (5%), and *C. tropicalis* (3%). A combination of different *Candida* species was found in three patients: (1) *C. tropicalis* and *C. parapsilosis* and *C. auris*; (2) *C. tropicalis* and *C. auris*; and (3) *C. albicans* and *C. parapsilosis*. 

The etiology of IC in patients with COVID-IC and IC without COVID differed (Table 2). Despite the fact that *C. albicans* remained the most common causative agent of invasive candidiasis, there was a significant increase in the number of infections caused by *C. auris*, *C. parapsilosis*, and *C. tropicalis*.

The MICs of antifungal drugs were determined for *Candida* species. Against *C. glabrata*, the MIC range of fluconazole was 16–32 mg/L, for voriconazole, it was 0.5–2 mg/L, and for amphotericin B, it was 0.125–0.5 mg/L; against *C. parapsilosis*, the MIC range of fluconazole was 0.5–2 mg/L, for voriconazole, it was 0.03–0.25 mg/L, and for amphotericin B, it was 0.125–0.25 mg/L; against *C. auris*, the MIC range of fluconazole was 64–256 mg/L, for voriconazole, it was 1–4 mg/L, for amphotericin B, it was 1–8 mg/L, and for caspofungin, it was 0.03–0.5 mg/L.

*C. albicans* was sensitive to azoles (fluconazole (100%), voriconazole (100%)) and echinocandin (caspofungin (100%)). *C. parapsilosis* was sensitive to azoles (fluconazole (90%), voriconazole (100%)), and amphotericin B (100%). 

*C. glabrata* was sensitive to voriconazole (70%) and amphotericin B (100%) and was resistant to fluconazole (100%). *C. auris* was resistant to azoles (fluconazole and voriconazole), amphotericin B, and sensitive to echinocardine (caspofungin) in 100% of cases.

### 3.5. Treatment and Outcome

**Figure 4:**
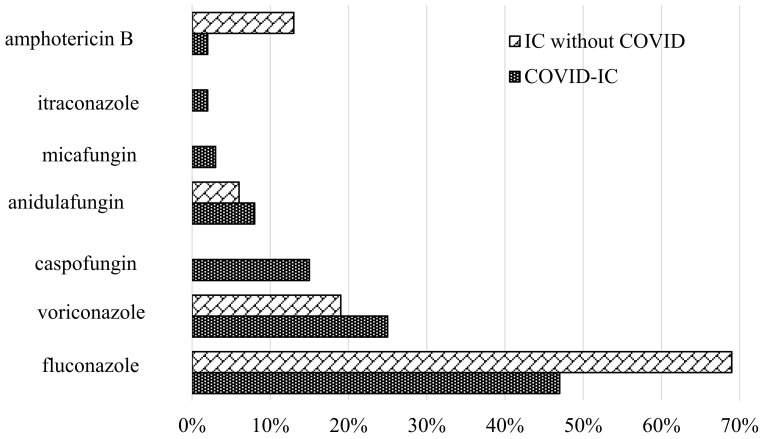
Antifungal therapy in the study and control groups.

Empirical (before the identification of *Candida* spp. with blood culture) therapy was used in 21% of COVID-IC patients. The initial drug for empirical therapy was fluconazole (93%). Echinocandin was used as an empirical therapy in 7% of cases. The majority (79%) of COVID-IC patients received antifungal therapy after laboratory confirmation of the diagnosis of IC. The prescribed therapy in COVID-IC patients and in the IC without COVID control group was compared. The main drugs used for the treatment of IC in both groups were azoles (fluconazole, 47% vs. 69%, and voriconazole, 25% vs. 19%). Echinocandin was more often used in COVID-IC patients (26% vs. 6%) (Figure 4). It should be noted that all patients with *C. auris* received echinocandin for directed therapy. 

The overall survival rate of the examined individuals within 30 days from the moment of diagnosis of COVID-IC was 45%, which was significantly lower than in patients with IC without COVID-19 (63%, *p* = 0.011). COVID-IC was a postmortem finding in four patients who did not receive antifungal therapy.

### 3.6. Analysis of Prognostic Factors

**Figure 5:**
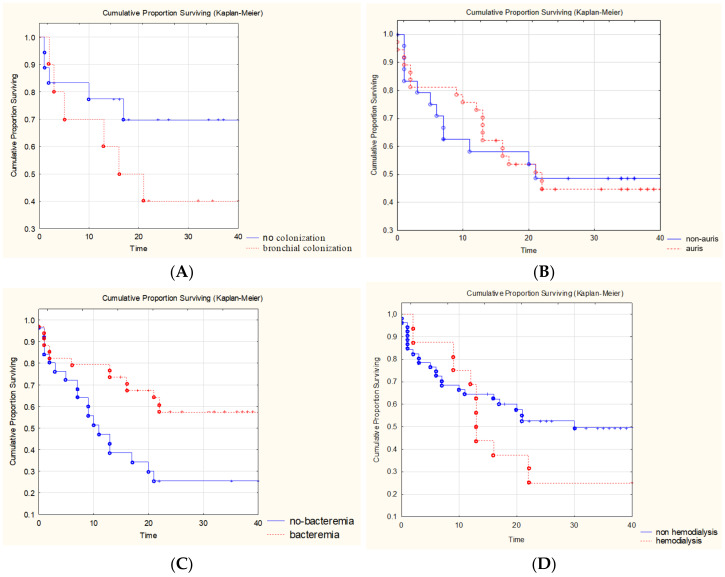

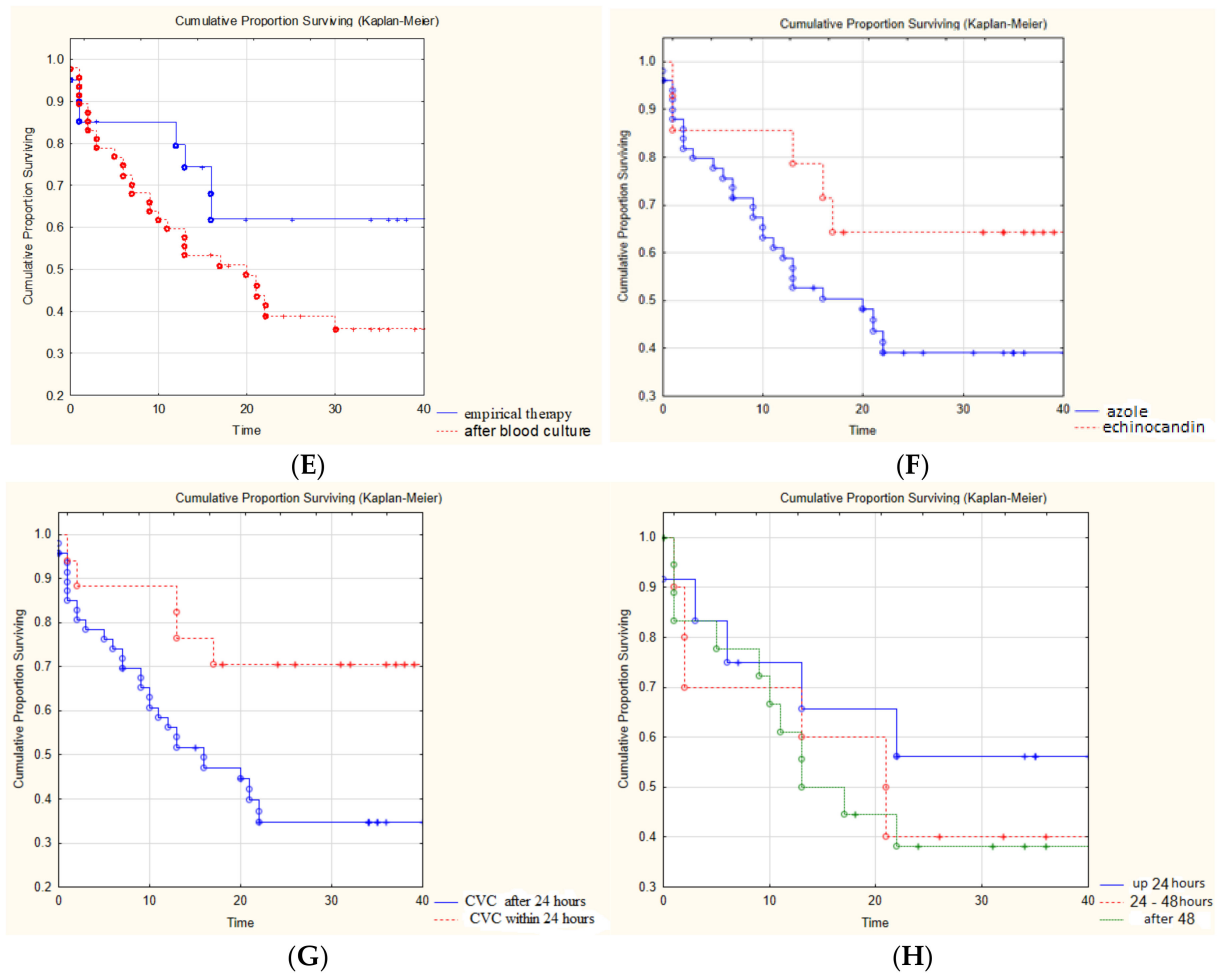
The influence of various factors on survival rate of patients with COVID-IC: (**A**) bronchial colonization by fungi of the genus *Candida*; (**B**) *C. auris* and non-*C. auris* infection; (**C**) concomitant bacteremia; (**D**) hemodialysis; (**E**) empirical therapy; (**F**) use of echinocandin; (**G**) change of central venous catheter; (**H**) initiation of the antifungal therapy.

The impact of various factors on the 30-day survival rate in patients with COVID-IC was evaluated in comparison with the IC without COVID control group (Figure 5). The reliable prognostically unfavorable factors in comparison with the IC without COVID control group were the presence of bronchial colonization by fungi of the genus *Candida* (40% vs. 70%) and concomitant bacteremia (26% vs. 57%). The survival rate was worsened by the long-term (more than 10 days) use of high doses of SGCs (in prednisone-equivalent doses of 0.5–4 mg/kg/day (median 1.65 (0.5–2.65) mg/kg/day)) (40% vs. 47%) and hemodialysis (25% vs. 52%). There were no differences in survival between the groups of patients with *C. auris* and non-*C. auris* infection. The survival rates were improved by empirical therapy (62% vs. 38%), early antifungal therapy within 24 h after IC diagnosis (56% vs. 38%), the use of echinocandin (64% vs. 39%), and the early change of CVC within 24 h after the detection of IC (71% vs. 35%).

## 4. Discussion

It is known that patients with severe COVID-19 have marked impairments of local (epithelial damage, inefficiency of the ciliary clearance, etc.) and systemic immunity (lymphocytopenia, CD4-cytopenia, etc.) due to the viral infection itself or due to the use of SGCs and immunosuppressants. Such disorders may be accompanied by bacterial and fungal superinfections, including IC [1,5,13,14,15]. Our study confirmed that IC occurred in patients who stayed in medical facilities for a long time. In patients with COVID-19 without IC, the duration of hospitalization was shorter. The average time from ICU admission until the development of IC in patients with COVID-19 was 15 days, which is less than the 18 days shown in the earlier ERA study, which included patients with IC without COVID-19 in the Russian Federation [3].

A Turkish study, which included 236 cases of IC (105 patients with COVID-19 and 131 without COVID-19), also showed that IC developed in a shorter length of hospital stay in the presence of COVID-19 (13 days vs. 27 days (*p* < 0.001) [16].

In our study, the patients were in the ICU for a long time and had a high SOFA score (8 (3.6–12.4) points), which mean that they were exposed to multiple risk factors of IC. Amir Arastehfar [9] suggested dividing the risk factors of COVID-IC into two groups: standard risk factors typical for severe ICU patients and new risk factors related to the course of the COVID-19 patient.

The first group included the most common risk factors: numerous catheters, including a CVC (74.5%); the long-term use of several broad-spectrum antibiotics (60.5%); decompensated diabetes mellitus; surgical interventions on abdominal organs; invasive examinations; and parenteral nutrition [5,9]. It is well known that catheters (including CVC) are the entrance gates of nosocomial *Candida* infection, especially for pathogens such as *C. auris* and *C. parapsilosis*. At the same time, azole-resistant *C. auris* and *C. parapsilosis* can persist in the hospital environment and on the devices and hands of medical personnel, and subsequently cause drug-resistant candidiasis and/or candidemia in patients who did not previously receive antifungal drugs [17,18]. According to our study, the presence of CVC for more than 10 days significantly increased the risk of developing IC (OR = 70 [15–309]) in patients with COVID-19 and occurred in 98% of our patients, which was comparable with the data of international studies (74–94%) [5,9,19].

An Italian group of experts (Gruppo Access Venosi Centrale a Lungo Termine; GAVECeLT) [20] suggested the use of additional measures to prevent catheter-associated infection, including *Candida* infection. They proposed the use of mainly peripheral catheters via femoral access to minimize the risk of infection in patients with oropharyngeal and tracheal secretions during catheter insertion and the administration of ultrasound control during the introduction of any central venous access.

All patients in our study received antibacterial therapy, mostly two or more antibacterial drugs at the time of the development of IC. Such antibacterial therapy could change the composition of the resident microbiota of the mucous membranes and increase the colonization of the intestinal wall via pathogenic microbiota. A number of studies indicated a link between the use of broad-spectrum antibiotics (more often vancomycin) and the occurrence of candidemia [21].

Patients with sepsis, which was observed in severe COVID-19 cases, could develop damage to the gastrointestinal mucosa integrity, which contributed to the transposition of *Candida* into the bloodstream [9,22]. According to our study, previous or concomitant bacteremia significantly increased the risk of developing IC in COVID-19 patients (OR = 10.6 [4.8–23.3]) and was found in 83% of patients, which was higher than the data in the literature (54%) [16]. Scientists from Israel [23] analyzed 444 cases of candidaemia (450 *Candida* isolates) and studied the effect of antibacterial and antimycotic therapy on *Candida* resistance. The authors indicated that candidemia caused by *C. glabrata* was closely related to recent exposure to metronidazole (OR = 3.2; *p* < 0.001). Infection with a fluconazole-resistant isolate was associated with exposure to carbapenems, trimethoprim-sulfamethoxazole, clindamycin, and colistin (OR = 2.8; *p* = 0.01). According to our data, a reliable risk factor for the development of COVID-IC was surgery on the abdominal organs over the past 2 weeks, which was noted in 20% of patients. In the literature, we did not find data about the frequency of this risk factor in COVID-IC patients.

The second group of risk factors included conditions related to COVID-19 infection. The severe course of COVID-19 was associated with the development of acute respiratory distress syndrome (ARDS). Therefore, such patients were on artificial pulmonary ventilation, which, according to our data, was a significant risk factor for the development of IC (OR = 12.9 [5.9–28.4]) and occurred in 79% of patients. According to the literature, *Candida* colonization of the respiratory tract was observed in 15–25% of patients with COVID-19 after 48 h of ventilation, and the duration of ventilation correlated with the percentage of colonization [9,24]. Clinical studies showed that the frequency of *Candida* isolation from the BAL in the ICU patients could reach 50%, which increased the median hospital stay (59.9 vs. 38.6 days, *p* = 0.006) and hospital mortality (34.2 vs. 21.0%, *p* = 0.003) compared to the patients without IC [25,26]. According to our study, colonization of the bronchial tree by fungi of the genus *Candida* is a prognostically unfavorable factor that worsens the survival of COVID-IC patients (35% vs. 75%). However, at the same time, in the practical recommendations, there were indications that antifungal therapy should not be used routinely in patients with a *Candida* airway colonization [27].

The limitation of our study was the wide confidence intervals for variables (e.g., OR for risk factors), which were probably due to the small sample size.

Our study showed that *C. albicans* was the most common species of yeast among patients with COVID-19 in critical condition (41%). Our findings were comparable with the data in the literature; *C. albicans* was identified in 44.1% of cases [9]. Another common *Candida* species in our study and Indian studies was *C. auris*, which was detected in 25% of cases [5]. We assessed the effect of the presence of *C. auris* on survival and did not find any differences from a non-*C. auris* infection.

In our study, we noted a low survival rate for 30 days in the patients with COVID-IC compared to the patients with IC without COVID-19 (45% vs. 63%). According to the literature, the mortality rate of the patients with concomitant fungal infections was 46–53%, which was much higher than in the patients without concomitant fungal infections (25.8–31%) [9,28]. Nevertheless, it was impossible to assess the degree of association of COVID-IC with the level of attributive survival of patients since most patients had concomitant severe conditions. We tried to identify the predictors that affected patient survival. The use of high doses of SGCs could have affected the development of invasive mycosis as they had an immunosuppressive effect on neutrophils, monocytes, and macrophages. In another study, it was indicated that treatment with corticosteroids was associated with a 3.33-fold increase in the development of concomitant fungal infections [29]. According to our data, the long-term use of high doses of SGCs reduced the survival rate of patients by 7%. We showed that hemodialysis significantly reduced the survival rate of our patients (26% vs. 51%). Furthermore, the prognostically unfavorable factors that worsened the survival of the patients with COVID-IC compared to the patients with IC without COVID-19 were the colonization of the bronchial tree by fungi of the genus *Candida* (35% vs. 75%) and concomitant bacteremia (26% vs. 75%).

The early diagnosis and treatment of candidiasis were key factors in improving the survival of patients with IC. The survival rate was increased by empirical therapy (66% vs. 44%), the early (within 24 h) prescription of antifungal therapy (56% vs. 38%), and the use of echinocandin (64% vs. 39%). However, in our study, echinocandin was used for the treatment of IC only in 26% of cases. The main focus for the prevention of IC should be related to medical interventions. We showed that an early change of CVC within 24 h after the detection of IC improved patient survival (71% vs. 35%).

## 5. Conclusions

Patients with COVID-19 had an increased risk of co-infection, including invasive candidiasis. The statistically significant risk factors were the presence of CVC for more than 10 days, artificial pulmonary ventilation for more than 11 days, concomitant bacteremia, hemodialysis, and surgical treatment performed during the previous 2 weeks. *Candida albicans* remained the leading causative agent of invasive candidiasis, but the number of azole-resistant pathogens such as *C. auris* grew. The overall survival rate of COVID-IC patients for 30 days was 45%. The factors that affected survival and worsened the prognosis were concomitant bacteremia, hemodialysis, long-term therapy with SGCs, and bronchial colonization with *Candida*. The survival prognosis was improved by the early change/replacement of CVC (within 24 h), the initiation of empirical therapy, and the use of echinocandin.

## Figures and Tables

**Table 1:** Characteristics of the study and control groups.

	COVID-IC	COVID-19 without IC	IC without COVID-19
number	72	75	78
gender, males/females	49%/51%	49%/51%	51%/49%
age, median, years (range)	61 (29–96)	63 (29–89)	60.5 (37–91)
SOFA score, Me (Q1-Q3)	8 (3.6–12.4)	7 (3.6–10.4)	6.5 (3.0–9.0)
types of invasive candidiasis	candidemia–65 pericardial–4 peritoneal–3	–	candidemia–70 pericardial–2 peritoneal–6

**Table 2:** Etiology of invasive candidiasis in COVID-IC vs. IC without COVID groups of patients.

*Candida Species*	COVID-IC, *n* (%)	IC without COVID, *n* (%)
*C. albicans*	31 (41%)	45 (56%)
*C. auris*	18 (23%)	1 (1%)
*C. parapsilosis*	6 (8%)	2 (3%)
*C. guilliermondi*	5 (7%)	1 (1%)
*C. glabrata*	4 (5%)	5 (6%)
*C. tropicalis*	2 (3%)	2 (3%)
*C. dubliniensis*	1 (1%)	1 (1%)
*C. krusei*	-	2 (3%)
*C. kefyr*	-	1 (1%)
*C. lusutaniae*	-	1 (1%)
not identified	9 (12%)	19 (24%)

*n*—number of *Candida* isolates.

## Data Availability

Data are available in the Department of Clinical Mycology, Allergy and Immunology, Kashkin Research Institute of Medical Mycology, North-Western State Medical University, named after I.I. Mechnikov, Saint-Petersburg, Russia.
